# Optical Fiber Sensors Based on Fiber Ring Laser Demodulation Technology

**DOI:** 10.3390/s18020505

**Published:** 2018-02-08

**Authors:** Wen-Ge Xie, Ya-Nan Zhang, Peng-Zhao Wang, Jian-Zhang Wang

**Affiliations:** 1 College of Information Science and Engineering, Northeastern University, Shenyang 110819, China; xiewenge@stumail.neu.edu.cn (W.-G.X.); wangpengzhao@stumail.neu.edu.cn (P.-Z.W.); wangjianzhang@stumail.neu.edu.cn (J.-Z.W.); 2 State Key Laboratory of Synthetical Automation for Process Industries, Shenyang 110819, China

**Keywords:** fiber ring laser, optical fiber sensor, Mach–Zehnder interferometer, Fabry–Perot interferometer, Sagnac, fiber Bragg grating

## Abstract

A review for optical fiber sensors based on fiber ring laser (FRL) demodulation technology is presented. The review focuses on the principles, main structures, and the sensing performances of different kinds of optical fiber sensors based on FRLs. First of all, the theory background of the sensors has been discussed. Secondly, four different types of sensors are described and compared, which includes Mach–Zehnder interferometer (MZI) typed sensors, Fabry–Perot interferometer (FPI) typed sensors, Sagnac typed sensors, and fiber Bragg grating (FBG) typed sensors. Typical studies and main properties of each type of sensors are presented. Thirdly, a comparison of different types of sensors are made. Finally, the existing problems and future research directions are pointed out and analyzed.

## 1. Introduction

Due to the demand of automobile industry, military aeronautics, electric system, oil exploration, environmental monitor and so on, sensor technology has attracted more and more attention of scholars from all over the world. The optical sensors have been investigated with increasing interest due to their advantages such as anti-interference, high sensitivity, remote sensing, multiplexing capabilities, compact size and low cost [1,2]. Optical fiber sensors have been widely used for monitoring and measuring of various parameters, such as refractive index (RI) [3,4,5], temperature [6,7,8], strain [9,10,11], bend [12,13] and so on.

Various optical fiber sensing schemes including Mach–Zehnder interferometer (MZI) [3,4,6,7], Fabry–Perot interferometer (FPI) [11], Sagnac [8,9], fiber Bragg grating (FBG) [10] have been proposed for these parameters. In the process of fabrication and application of these sensors, there may exist some problems for reading the data of output spectra, such as big loss, wide bandwidth, low signal-to-noise radio (SNR) and burrs, which can cause reading errors for the resonance wavelength. Therefore, some demodulation methods have been proposed. Phase generated carrier [14], heterodyne [15] and 3 × 3 couple demodulation method [16] are the common demodulation techniques. Compared with these demodulation methods, the demodulation system based on fiber ring laser (FRL) is simple, accurate and easy to operate.

In this paper, the applications of the FRL demodulation technology in optical fiber sensors are described in detail, including principle, application, prospect and development. The principles of the FRL are described and analyzed in Section 2. Then in Section 3, various application methods classified according to the principle of sensing are presented and the advantages and disadvantages of the application methods are compared. Finally, the future directions and conclusion of the FRL are proposed in Section 4.

## 2. Principle

**Figure 1:**
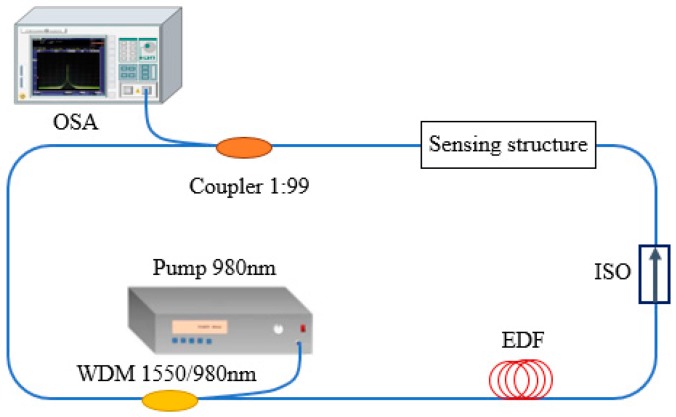
The schematic diagram of fiber ring laser (FRL).

The schematic diagram of FRL is shown in Figure 1. The FRL consists of a section of erbium-doped fiber (EDF), a wavelength division multiplexer (WDM), an isolator for ensure unidirectional propagation of light, a coupler for coupling the light of the cavity, a 980 nm pumping source for exiting laser and a sensing structure which is acted as the sensing head and the band-pass filter of the FLR system. The tailorable filtering structures include MZI, FPI, Sagnac, FBG and so on. The WDM for 1550 nm and 980 nm is used to combine and divide the light with different wavelength. The EDF is used as an active medium which can be pumped by a 980 nm diode laser and its signal wavelength ranges from 1520 to 1565 nm [17]. When light emitted from the pump source transmits through EDF, it will occur stimulated emission. After recycling in the ring cavity, laser spectrum will be recorded by an optical spectrum analyzer (OSA). When the filtering structure, such as MZI, is embedded into the FRL device as sensing part, the sine-shaped interference spectrum acts as a wavelength selector and only one peak is amplified to become the laser line. It means that the laser wavelength will move along with the wavelength shift of filter. When the interference spectrum of the MZI shifts with the variation of external parameters, such as temperature, strain and refractive index, the laser wavelength will shift also along with these external parameters, which is the measurement principle of the FLR based sensors. 

**Figure 2:**
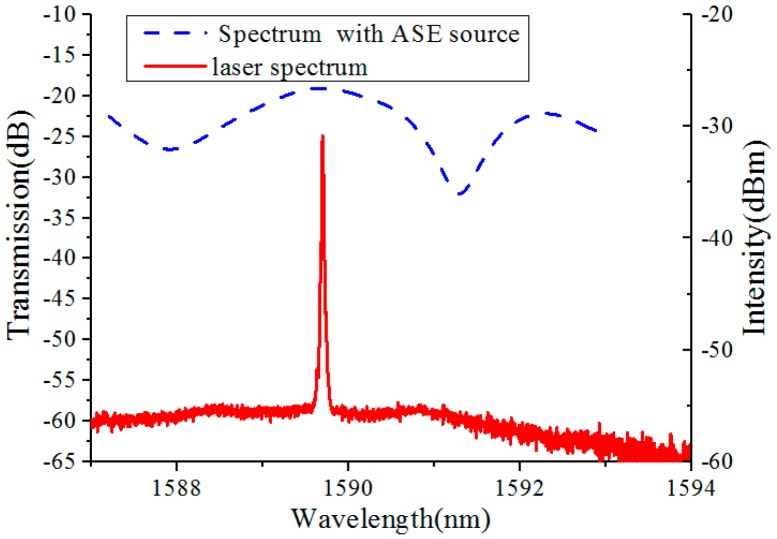
The comparison between the spectrum with amplified spontaneous emission (ASE) source (blue line) and laser spectrum (red line) after demodulated by the FRL.

The comparison between the spectrum with amplified spontaneous emission (ASE) source and laser spectrum after demodulated by the FRL is shown in Figure 2. It can be seen that the laser spectrum has high intensity, narrow full width at half maximum (FWHM) and high SNR. Therefore, the spectral quality and the sensing detection limit of the sensor structure are improved. The DL (Detection Limit) is expressed as:

(1) $$DL=\frac{R}{S}$$

where *S* is the measurement sensitivity and *R* is sensor resolution. *R* is mainly determined by *σ*_ampl-noise_, *σ*_temp-induced_, and *σ*_spect-tes_, which respectively stand for the amplitude noise, thermal variation and the spectral resolution of the system setup [18]. The resulting spectral variation can be approximated by:

(2) $$\sigma_{\mathrm{ampl}-\mathrm{noise}}\approx (FWHM)/(4.5\times {\left(SNR\right)}^{0.25})$$

where *SNR* is complex to be measured in spectrum and the FWHMs are respectively the interference spectrum and laser spectrum in experiments. The sensor resolution in the system is used as:

(3) $$R=3\sigma =3\sqrt{\sigma_{\mathrm{ampl}-\mathrm{noise}}^{2}+\sigma_{\mathrm{temp}-\mathrm{induced}}^{2}+\sigma_{\mathrm{spect}-\mathrm{tes}}^{2}}$$

The temperature stabilization of *σ*_temp-induced_ is generally small enough to be ignored and the $\sigma_{\mathrm{spect}-\mathrm{tes}}=R_{w}/2\sqrt{3}$ in which the *R*_w_ is the wavelength scanning resolution of OSA. According to Equations (1)–(3), the sensor resolution before and after combining with the FRL can be calculated. The sensitivity is not change but the DL is greatly improved [19].

## 3. Sensing Applications

### 3.1. Mach–Zehnder Interferometer Typed Sensors

The MZI is formed by the interference of two beam light, which propagate on two different paths. Light from the light source will be divided into two beams and spread into different paths. When the two beams couple at one point, they will interfere with each other, and interference intensity can be expressed by [20]:

(4) $$I=I_{1}+I_{2}+2\sqrt{I_{1}I_{2}}\cos(\varphi +\delta )$$

where *I*_1_ and *I*_2_ are the light intensities of the two interference beams, *φ* is phase difference of the two paths, δ is the initial phase, the phase difference can be expressed as:

(5) $$\varphi =\frac{2\pi (n_{\mathrm{eff}1}-n_{\mathrm{eff}2})L}{\lambda }=\frac{2\pi (\Delta n_{\mathrm{eff}}L)}{\lambda }$$

where *n*_eff1_ and *n*_eff2_ are the refractive indices of two paths, *L* is the length of interference region, *λ* is the operating wavelength of input light. In the system, the MZI acts as a filter and sensing unit which feels the changes in the external environment.

In the past few years, the MZI typed sensors has been widely studied. Comparing to other types of optical fiber sensors, the MZI typed sensors are well-known for their good qualities of low cost, robustness, simple structure and easy fabrication process, which make them suitable for various applications, like temperature, RI, strain, and so on. However, researchers also noticed that the conventional MZI typed sensing systems illuminated by broadband light sources [21,22,23,24,25] have to suffer the problem of low measurement resolution and a big difficulty in remote sensing. Recently, the focus on the combination of FRL and MZI find a new way to solve such problems. The high SNR and narrow 3-dB bandwidth that FRL brings into the sensing system make the co-existence of high sensitivity and remote sensing possible. Also, the narrow 3-dB bandwidth will enhance the resolution of the sensing system significantly, which assures the sensors to have the ability of precision sensing.

#### 3.1.1. Temperature Sensing

**Figure 3:**
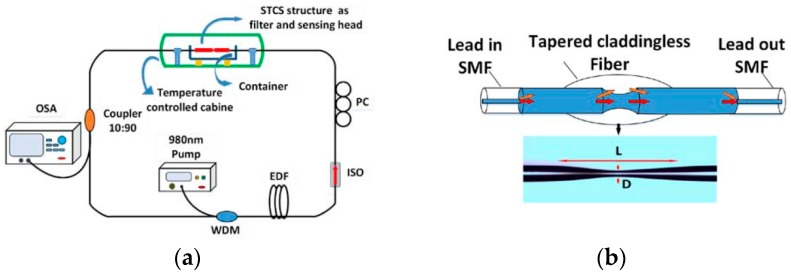
(**a**) Experimental set up of the laser sensing system based on single-mode-tapered claddingless-single-mode (STCS); (**b**) Schematic configuration of STCS fiber structure.

In 2014, L. J. Liang et al. [26] proposed a novel refractive index (RI) and temperature all-fiber ring sensor based on single-mode-tapered claddingless-single-mode (STCS) fiber structure as shown in Figure 3a. The STCS fiber structure was fabricated by a section of tapered claddingless fiber (TCF) which was spliced between two sections of standard single mode fiber (SMF) as shown in Figure 3b. The STCS fiber structure acted as the sensing head and filter of the laser system simultaneously. This multimode interference (MMI) based on fiber laser sensing system enhanced the SNR and narrowed the 3-dB bandwidth significantly. According to the experimental results, average sensitivity of the sensor reached 163.80 nm/RIU in the RI range of 1.333–1.399 and a sensitivity of 10.8 pm/°C was achieved with temperature ranging from 8 °C to 80 °C. High optical SNR (~40 dB), narrow 3-dB bandwidth (<0.12 nm) and high resolution were also achieved due to this laser sensing system. The easy fabrication and the wide temperature measurement range make the sensor more applicable to most of the real environment, however, its sensitivity still needs to be improved and the tapered structure may make the sensor more vulnerable.

The next year, a significant effort was made to enhance the sensitivity of this kind sensors by Martinez-Ríos et al. [27]. They presented an EDF laser temperature sensor with an intracavity fiber filter immersed in glycerol/water solutions acting as a sensing element. The sensing head was actually a MZI based on a two-taper fiber. The two-tapered structure and the immersing of the MZI into glycerol/water solution of 98/2 (wt %/wt %) enhanced the sensitivity of the sensor enormously and made it finally reached a level of 1089 pm/°C. The FRL system used in this sensor brought a SNR of 50 dB. With all these modification on the conventional MZI and FRL structure to improve the sensitivity, the research group still controlled the fabrication process in a low complexity level, which made the sensor less costly. However, the trade-off between the sensitivity and temperature measuring range of this sensor made its application has to be limited in a higher temperature range of about 55~70 °C, which reduced its scope of application somehow. We also cannot neglect that, the two-tapered structure also can decrease the tensile strength of the sensor notably, which may also limit the application width of the sensor.

In 2016, to ensure the robustness and tensile strength, Cai et al. [19] simply used a common fusion splicer to fabricated two offsets with a short distance of 3.7 µm to constitute an offset MZI which is inserted into an Erbium doped FRL to act as a sensing element as well as a filter. The combining of the MZI and the EDF ring cavity laser improved the detection limits of the temperature sensor to 0.35 °C. Apart from that, the spectral quality factor Q also experienced a significant elevation. The core-offset structure showed its merits of low cost and easy fabrication process, and the fiber ring cavity laser structure enhanced the SNR of the sensor to 60 dB (estimated value), which is almost the highest value that the MZI based sensors can gain. However, the research group paid little attention on improving the sensitivity of the sensor, which made the sensor gained a relatively low sensitivity of 0.049 nm/°C.

**Figure 4:**
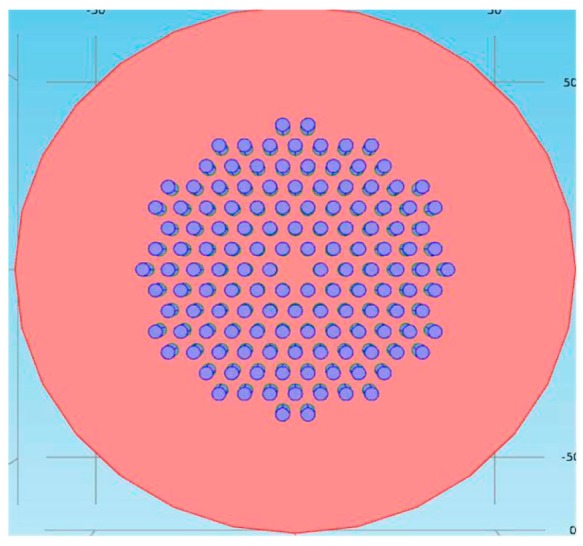
Schematic of the liquid-filled photonic crystal fiber (PCF) (the red region is PCF and blue region is toluene).

Then in 2017, Yang et al. [28] presented a temperature sensor based on a fiber ring laser embedded with liquid-filled photonic crystal fiber (PCF). To fabricate the sensing head, they infiltrated some high refractive index (RI) liquid into an index-guiding PCF, which was also used to generate band gap-like effect and used as the laser filter simultaneously. LMA-8 PCF was the main material used to build the sensing head due to its special structure of its cladding which contains six layer air holes. As shown in Figure 4, liquid with high RI was infiltrated into the air holes of the PCF. In the experiment, the emission wavelength of the fiber laser ring shifted to the shorter wavelength with an increasing of the output intensity when the temperature was increased. As a result, the sensor gained a temperature sensitivity of −1.747 nm/°C and 0.137 dB/°C. The measured SNR of the sensor could be 55 dB while the 3-dB bandwidth was less than 0.08 nm due to the insertion of FRL. The sensitivity of this sensor reached a new level among almost all of the MZI typed temperature sensors combined with FRL, however, the sacrifice of the measurement range should also be noticed. The measurement ranged from 25 °C to 31 °C was not only too narrow in real sensing environment, but also too confined in a low temperature scope. So, the application environment of the sensor should be carefully chosen to make its high sensitivity show the value of it.

#### 3.1.2. Refractive Index Sensing

MZI typed sensors are largely used in the field of RI sensing due to its excellent qualities of low cost, simple structure and easy of fabrication. To solve the problem in most of the conventional sensing systems which use ASE as the light source and OSA as the monitor that the resolution is low and the 3-dB bandwidth is high, scientists focus on the combination of FRL and the MZI structures.

In the year of 2014, Liu et al. [29] realized that though the application of the combination of FRL and MZI-based sensors has been widely discussed, the introduction of them to the measurement of RI has not been deeply researched. So, in the willing to provide a low-cost sensing system with high resolution and the ability of remote sensing to detect RI, they presented a singlemode–claddingless–singlemode (SCS) fiber structure based on fiber ring cavity laser in 2014. The SCS fiber structure of the FRL acted not only the filter but also the sensing head. This special structure ensured the sensor to gain a good linearity and finally reached a sensitivity of ~131.64 nm/RIU when the aqueous solution RI ranging from 1.333 to 1.3707. They also promoted the SNR of the sensor to 50 dB, and narrowed the 3-dB bandwidth to less than 0.03 nm. The extraordinary high linear quality of the sensor which is higher than 0.999 assured its performance in small range measurement applications.

**Figure 5:**
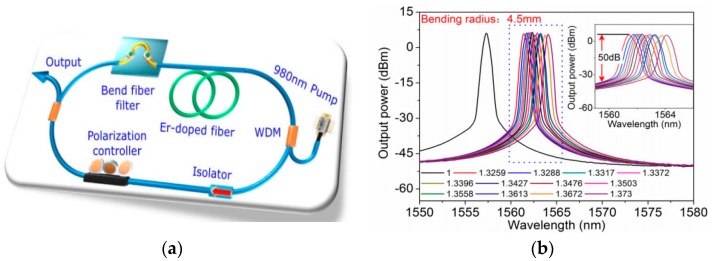
(**a**) Schematic of the proposed FRL; (**b**) Red shift of the fringes as the refractive index (RI) increases.

By investigating the work that have been made in the area of RI sensing based on optical fiber systems, X. P. Zhang et al. [30], similar to Liu et al. [29], realized that though most of the RI sensors can gain a high sensitivity when they apply to the experimental environment, their undue sophisticated structure and demodulation schemes always make them too expensive to be adopted into some of the real environment. Meanwhile, they also suffer the problem of having a wide bandwidth, which can harm their sensing precision. So, a novel RI sensor based on a FRL incorporating a bent fiber filter was proposed by his research group in 2015, as showed in Figure 5a. The bent fiber filter consisted of a section of semicircular bare standard SMF with a selected bending radius. At the input end of the semicircular bare SMF, the cladding modes were excited due to the bending and interact with the surrounding during the propagation along the bare fiber. Then, the cladding modes recoupled back to the core mode at the terminal of the semicircular bare SMF, and all cladding modes interfered with the core mode. Therefore, the sensing structure formed a novel MZI interferometer. Figure 5b showed the measured laser output spectra with different RI and the magnified laser output spectrum with an output power of 5.8 dBm, high SNB of 50 dB and 3 dB bandwidth less than 0.3 nm. Experimental results show the proposed sensor had a red wavelength shift with an increasing RI, and the maximum sensitivity was 124 nm/RIU when a 4 mm bending radius was selected. It can be seen that with an easy fabrication of the sensing system, the sensor still maintained a relatively high sensitivity.

Inspired by the RI sensor that is presented by Yao et al. [31] and determined to perfect their research, Xing et al. [32] inserted a segment of cladding-less fiber into two sections of SMF with on core-offset joint in order to enhance the extinction ratio of their RI sensor in 2016. With the help of the all-FRL sensing system, the sensor achieved a RI sensitivity of 52.3 nm/RIU in the RI range of 1.334–1.370 as well as a 3-dB bandwidth below 0.2 nm and an optical signal-to-noise ratio near 30 dB. Compared to the work of Yao et al. Xing et al. did simplify sensing structure and gain a higher sensitivity. 

Then in 2017, different from most of the research groups focusing on using the multimode fiber (MMF) in RI sensing, Liu et al. [33] realized the shortcoming of the MMF that the modal interference excited by too many core modes in MMF makes it hard to provide a smooth comb spectrum to select the lasing wavelengths. So, the attention on the few-mode fiber (FMF) inspired him to present a RI sensor based on a special few-mode-single mode-few-mode (FSF) fiber structure. The few-mode fiber was a concentric ring core fiber which can support only two scalar modes. They used the stable mode interference, which would occur in the sensing system, as a sensing head and a filter to select the lasing wavelength. A sensitivity of 45.429 nm/RIU can be reached in the range of 1.333–1.363 with the optical signal to noise ratio of 45 dB and narrow 3-dB bandwidth of 0.1 nm. By comparing to the traditional multimode-single mode-multimode (MSM) fiber structure, the FSF structure did solve the problem that the spectrum response of MSM structure is uncontrollable. However, it is also found that the sensitivity of this sensor is relatively low comparing to other MZI typed sensors based on FRL structure.

#### 3.1.3. Strain Sensing

In 2014, Bai et al. [34] realized the low thermos-optical coefficient of PCF can avoid the cross-sensitivity problem between strain and temperature when applied in MZI typed strain sensors, and innovatively combined the PCF structure with FRL to enhance the SNR and narrow the 3-dB bandwidth of their new strain sensor. So, they spliced a piece of PCF between two SMFs to make a PCF in-line MZI structure (PMS) which served as an optical band-pass filter and the sensing head based on fiber ring cavity laser. This sensor reached a sensitivity of 2.1 pm/με in the linear strain range of 0–2100 με. Comparing to the conventional PMS strain sensors, the sensitivity of 2.1 pm/με is relatively high. Apart from that, its wide measurement range also made its application more wide-open. However, the sensitivity of the sensor still has room for improvement.

Few months later, seeing the shortage in the sensitivity of other strain sensors based on optical fiber structure [35,36,37], Pei et al. [38] demonstrated a highly sensitive axial strain fiber laser sensor based on all-fiber acoustooptic tunable filter (AOTF). The all-fiber AOTF was used as the sensing head for axial strain measurement. The recording of the central wavelength of the sensor which is changed by the effect of the axial strain made the measurement become possible. It was tested that this sensor can gain a high sensitivity of about 0.148 nm/με in the range of 0–160 με with a narrow bandwidth (~0.05 nm) and a high optical signal-to-noise ratio (~40 dB). The promotion of the sensitivity did not bring a complex structure with difficult fabrication process. The fabrication of AOTF is quite simple in their experiment.

Like what Bai et al. [34] did, Chen et al. [39] also paid a lot attention to reduce the influences of temperature changes on strain sensing. By using silica fiber which has a low thermal expansion coefficient and construct the stretched abrupt-tapered micro-fiber (SAT-MF) structure, they demonstrated a temperature-insensitive strain sensor base on SAT-MF in the erbium fiber laser ring cavity which achieved their goal. The abrupt-tapered fiber was made by using a micro hydrogen flame to heat and stretch a SMF and finally gained a diameter of 58.7 μm. Under this circumstance, the strain sensitivity of this sensor can achieve above 4.443 nm/mε. Maintaining such a considerable sensitivity under an impressive measurement range of 0–7.0 mε while the temperature dependence is controlled in a low level, this sensor showed its merit in industrial applications. However, it also has to be noticed that the tapered structure may cause some intensity problem to this sensor.

Kang et al. [40] also noticed that reducing the cross-sensitivity between the measurands is a major problem in strain sensing. Different from the above two researchers, Kang found that little effort was made to describe the possibility of simultaneous measurement based on FRL sensors, and presented a novel laser sensing configuration for both strain and temperature in 2015. Than a decoupling method was proposed to detach the interaction between temperature and strain. A twin-core fiber (TCF) was embedded between a segment of SMF and a segment of FBG-written photosensitive SMF to fabricate a coupling comb filter. This whole structure is instated into an erbium doped FRL. The TCF part showed a good quality of its low insertion loss as well as the insensitivity to ambient humidity and RI. This sensor finally reached an optical signal-to-noise ratio higher than 40 dB, and a power instability of 6.75 µW in 1 h. The peak power sensitivities to strain reached 7.52 × 10^−4^ mW/με. The effort that Kang did to decouple the measurement of strain and temperature is innovative and inspirational.

**Figure 6:**
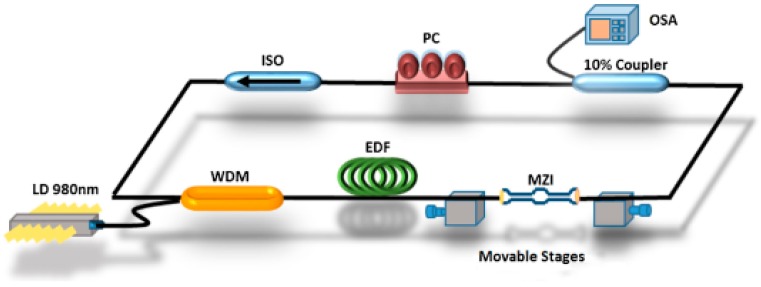
Experimental set up of strain sensor based on FRL.

In 2016, Jaddoa et al. [41] found the potential of tunable single wavelength fiber laser, and introduced an in-line Mach–Zehnder interferometer (IMZI) module with a waist diameter of 10 µm and a total length of 38 mm as a tuning device into an erbium-doped FRL to detect the strain. The whole experimental setup is shown in Figure 6.

The light will spill to two kinds of modes when it passing through the IMZI. The first is guided modes which can be guided to continue its propagation in the core of the fiber. The second part is the unguided modes which can interact with the ambient environment though the cladding by evanescent field. There will be some shifting in the wavelength of the light of unguided mode caused by the propagation delay in its interaction process with the ambient mediums. An interference signal can be realized and recorded since the IMZI devices let the two different modes of light recombining in the interferometer region. By recording the interference shifting, the volume of strain can be measured. As a conclusion, this sensitivity of this sensor reached 103.5 pm/µm, and a total tuning range of 6.19 nm that covers the wavelength from 1552.94 nm to 1559.13 nm was recorded. The wide tuning range of the sensor made it very adaptable for optical communication in Dense Wavelength Division Multiplexing (DWDM) and Coarse Wavelength Division Multiplexer (CWDM).

More recently, in 2017, Liu et al. [42] also introduced the few mode fiber into the strain sensing area, and demonstrated a few-mode concentric-ring core fiber (FM-CRCF) for axial micro-strain measurement with a FRL of a few-mode-single mode-few-mode (FSF) fiber structure which was used as an optical band-pass filter. Meanwhile, two sections of the CRCF served as the mode generator and coupler while the center SMF is the crucial part for sensing process. By constructing this special structure, they can gain a cleaner spectrum, which made it easier to measure the change of lasing peak wavelength. The combination of FSF and FRL gave this sensors sensitivity of 0.81 pm/με in a range from 0 to 1467 με with a 3-dB bandwidth less than 0.1 nm and a SNR of 45 dB. Such a wide measurement range with a considerable sensitivity showed the potential of this sensor in a wide application field, but the difficulty in its fabrication process may also limit its popularization.

#### 3.1.4. Liquid Level Sensing

**Figure 7:**
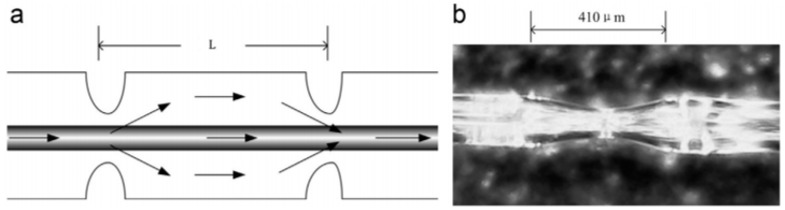
(**a**) Schematic diagram of the interferometer and (**b**) the microscope image of the taper.

Zhang et al. [43] in the year of 2014, pointed out that the precise measurement of liquid level should detach the influence brought by the temperature. Meanwhile, they also recognized that the fiber laser-based sensors can gain high sensitive measurement of liquid level. So, the research group designed a novel structure of a fiber interferometer with two tapers on it and combined it with a FBG to make a fiber laser sensor for simultaneous measurement of liquid level and temperature. A section of SMF (8.2 μm/125 μm) was used as the basic material for the fabrication of the interferometer. With a discharging for 1350 ms with intensity of 170 bit in the fiber fusion splicer, one taper could be realized on the SMF with a length of 410 μm and a diameter of 60 μm. Same method was used to fabricate the second taper. The schematic diagram of the interferometer and the microscope image of the taper is shown in Figure 7.

When this structure was implemented in the experimental process, the middle SMF showed its function as the sensing head, while the first taper and the second taper acted as the mode couplers to make the input light coupled to the core and cladding of the SMF. When it was aggregated with the FBG, the researching group accomplished their goal of measuring the liquid level and the temperature simultaneously, and finally reached the sensitivity of 0.2294 nm/mm in the range of 0–20 mm, and 0.0123 nm/°C in the range of 20–70 °C, respectively. The sensor also gained a high SNR of 50 dB. The sensitivity of this sensor was high enough for some precise measurement, however, the tapered structure may cause some vulnerability to this sensor, and the low repeatability and success rate in producing two tapers that are exactly same may increase the difficulty in its fabrication.

Two years later, knowing the difficulties in fabricating liquid level sensors based on optical fiber, Wang et al. [44] presented an easy-making and low-cost reflective liquid level sensor based on FRL with a single-mode-offset coreless-single-mode (SOCS) fiber structure. The ending-reflecting structure simplified the fabrication process of this sensor. In the laser ring system, the SOCS acted as the sensing head and laser filter. With the enhancement that the offset fusion brought to the sensor, the sensitivity of it reached 44.87 pm/nm, 61.60 pm/nm, 73.60 pm/mm and 86.27 pm/nm with the RI of 1.333, 1.353, 1.373 and 1.393, respectively. The FRL structure narrowed the 3-dB bandwidth of the sensor to less than 0.15 nm with a SNR of 30 dB.

#### 3.1.5. Magnetic Field Sensing

Bai et al. [45] investigated some of the traditional magnetic field sensing systems that combining magnetic fluid (MF) with some other optical structures like PCF [46], singlemode-multimode-singlemode (SMS) [47] fiber, fiber grating [48], and pointed out that the low visibility and 3-dB bandwidth of these sensors should be avoided. So, by introduced the FRL structure in, they made some improvement on the conventional structure. They built a single mode-no core-single mode (SNCS) fiber structure with a MF coating in 2016, and inserted it into a FRL cavity to realize magnetic field measurement. The special structure of the MF-coated SNCS fiber was used to be the sensing head and the band pass filter of the whole system. Thanks to the self-imaging effect occurred in their sensor during the sensing process, a high side-mode suppression ratio of 14 dB and a small insertion loss of around of −1.03 dB was achieved. Meanwhile, the sensor reached a magnetic field sensing sensitivity of 12.05 pm/Oe within the range of 15.9–222.32 Oe.

**Figure 8:**
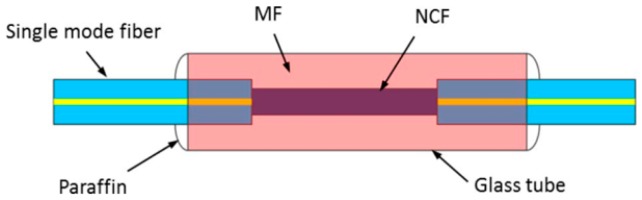
Schematic configuration of the structure of the sensing head of the remote magnetic field sensor based on intracavity absorption of evanescent field.

A similar sensing structure, but with the introducing of the intracavity absorption of evanescent field into the whole system as a measuring method, Shi et al. [49] proposed a novel sensor to detect the magnetic field in 2016. Like what Bai did, a MF-deposited SNCS fiber was also inserted into a FRL and worked as the filter and sensing head of the sensor. To make this structure, a piece of no-core fiber (NCF) was spliced between two SMFs. The MF was coated on a glass tube with both ends wrapped with paraffin, which was used to contain the combination of the NCF and two SMFs. The schematic configuration of the structure is shown in Figure 8.

Considering the evanescent field theory, the absorption of evanescent field would be induced in the interface of NCF and MF when the light propagating in the NCF. While the magnetic field of the ambient environment changed, the transmission spectrum and the transmission intensity would all be changed, which can be monitored and used to establish a relationship with the magnetic field of the surrounding. They finally gained a magnetic sensitivity of 52.1 pm/mT and −0.3679 dBm/mT with a high signal-to-noise ration of ~40 dB, and a narrow 3-dB bandwidth that is less than 0.05 nm.

#### 3.1.6. Other Domains of Sensing

Also, seeing the potential that FRL can bring in remote sensing, Sun et al. [50] proposed a FRL curvature sensor based on a SNCS structure which is simply fabricated by a fiber fusion splicer in 2016. The SNCS structure was used as the sensing element and the bandpass filter, and made the sensor gain a sensitivity of curvature of about −22.33 nm/m^−1^ in the range of 0.2121–0.3463 m^−1^.

When it comes to the measurement of gas concentration, a PCF-based spectroscopic absorption sensing platform is proposed by Zheng et al. [51], in the year of 2016. A piece of PCF and a long-period grating (LPG) was aggregate by the group and inserted in to a tunable erbium-doped FRL to build up their sensor. In the experimental atmosphere of NH_3_ with multiple gases combined, the sensor achieved a sensitivity of 17.3 nW/ppm.

In 2016, Liu et al. [52] decided to introduce the FRL into the displacement measurement. They tied a SMF loop into an erbium-doped FRL to achieve their goal. The SMF loop was used to be the sensing head and the filter of the sensor. The average sensitivity of their sensor reached 227.5 pm/mm in the displacement range of 0–30 mm.

In 2015, Hui Xiong et al. [53] proposed a sandwich structure of single mode fiber-thin core fiber-single mode fiber inserting in a FRL cavity, which formed a fiber laser bend sensor. The MZI was formed by a thin core fiber (TCF) sandwiched between two sections of SMFs. It could be seen that the sensor was temperature insensitive and had a large output light intensity ~0.61 dBm, a high SNB of ~63 dB, a narrow spectral width of ~0.01 nm and a good sensitivity of ~1.04 nm/m^−1^ within a curvature range from 0.8 to 2.0 m^−1^.

An overview of these various types of sensors based on MZI is presented in Table 1.

### 3.2. Fabry–Perot Interferometer Typed Sensors

The optical fiber FPI can be approximately equivalent to a two-beam interferometer and the FPI acted as a reflective construction. The reflective optical intensity can also be expressed as:

(6) $$I=I_{1}+I_{2}+2\sqrt{I_{1}I_{2}}\cos\varphi$$

which is similar to Equation (4), but the *I*_1_ and *I*_2_ are the intensities of the two reflected beams, respectively, and $\varphi =4\pi n_{\mathrm{eff}}L/\lambda$ is the phase difference of the two reflected beams. Comparing to some typical MZI typed sensors, FPI typed sensors can gain a relatively higher sensitivity by a simpler structure, which makes FPI typed sensors one of the classic optical fiber sensors. However, the FPI sensors also shared the same problem with MZI based sensors that conventional FP typed sensors also have a wide operating spectrum, which makes it difficult to promote their measurement accuracy. And the low SNR of some conventional sensors also limited their potential for remote sensing. Fortunately, more and more researchers are focus on solving this problem, and by their researching, the introducing of FRL into FPI based sensing systems can be a proper solution.

In 2016, Shi et al. [54] indicated that conventional gas pressure sensors based on optical fibers all suffered the limitation in remote sensing. Apart from that, the sensitivity that these sensors can gain are relatively low. So, the FRL structure was adopted by them, and firstly proposed a remote gas pressure sensor based on that. In their experiment, they inserted a FPI into a fiber laser working as a sensing element in gas pressure detection. This sensor based on a FRL embedded with a FPI and a Sagnac loop showed a transcendent quality in suppressing the wavelength competition due to the work of the fiber-optic Sagnac loop they used as filter in this FRL. The sensitivity of this sensor was promoted to −9.69 nm/kPa, and the 3-dB bandwidth was narrowed down to less than 0.02 nm with a high signal-to-noise ratio (SNR) ~45 dB.

**Figure 9:**
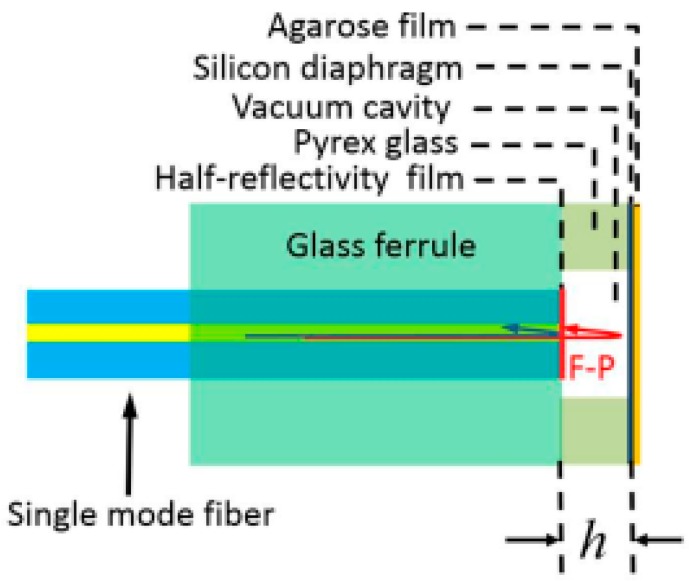
Schematic configuration of the humidity-sensitive FPI.

A year later, Shi et al. [55] introduced the method of combining the FRL and FPI into the area of humidity measuring. According to their paper, though the traditional fiber-optic humidity sensors have the merit of high sensitivity and anti-electromagnetic interference, most of them are lack of remote detection ability due to the broadband light source used in their sensing system which will cause a wide bandwidth and low peak intensity. The employ of FRL will fix that problem well. This designing thought inspired them to propose a humidity sensor based on FPI and intracavity sensing of fiber laser. They first built a FPI which is sensitive to humidity, and then inserted it into a FRL to induce intracavity humidity sensing. The humidity-sensitive FPI is shown in Figure 9.

**Figure 10:**
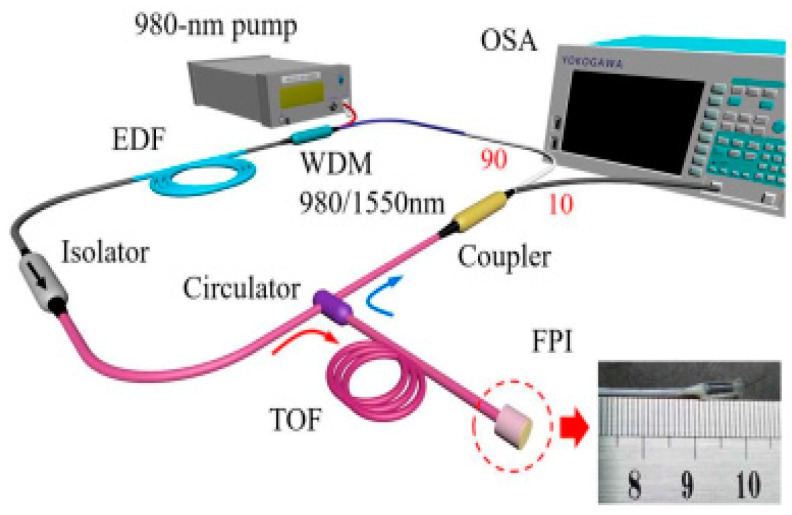
Experimental set up of humidity sensor.

To produce the Fabry–Perot (FP) effect, they sandwiched a hollow Pyrex glass between a SMF which is embedded in a glass ferrule with a half-reflectivity film coating on its end and a silicon diaphragm coated with Agarose film. The vacuum cavity with two reflective surfaces and a certain length that formed by this structure will cause the FP effect in the FPI. Because Agarose is sensitive to humidity, its RI will change with the ambient humidity, which means the FPI can be used for humidity detection. The whole experimental setup is shown in Figure 10.

An EDF of 3 meters was pumped by a 976-nm diode laser through a wavelength division multiplexer coupler. The FPI was inserted into the FRL by a circulator and transmission optical fiber (TOF). It was used as both the selective filter and the sensing head for humidity sensing. By combining the FRL and FPI, they finally promoted the SNR of this sensor to 45 dB, and narrowed its 3-dB bandwidth to less than 0.05 nm. The sensitivity of the sensor was 0.202 dB/%RH within the humidity resolution ranging from 20% RH to 98% RH.

In 2018, in order to further increase the measurement accuracy of temperature, Zou et al. [56] also used the method of inserting FRL into a FP-based sensing system, and presented a FRL sensor based on FP cavity interferometer for temperature sensing. They inserted a FP air cavity containing a segment of glass capillary between two SMFs to use it as the sensing element and filter. A moving towards longer wavelength will exist in the reflection spectra of the FP sensor when the temperature is increasing, and that can be monitored to measure the temperature of environment. In the experiment, a high temperature sensitivity of 0.249 nm/°C was gained.

An overview of these various types of sensors based on FPI is presented in Table 2.

### 3.3. Sagnac Typed Sensors

The Sagnac typed sensors are designed based on the Sagnac effect, also called fiber loop mirror, which is a phenomenon encountered in interferometry that is elicited by rotation. Normally, a typical Sagnac interferometer is a construction of a fiber loop with a section of birefringent fiber inserted into it. The birefringent fiber is used to make a path difference and a phase difference of the two counter-propagating waves to cause the interference effect that can be monitored on a spectrograph. The output of the Sagnac interferometer can be described as [57]:

(7) $$I=[1-\cos(\frac{2\pi BL}{\lambda })]/2$$

where *B* is the RI volume shifting from the fast axis to the slow axis of the birefringent fiber, *L* is the length of the birefringent fiber, and λ is the wavelength of the free-space light beam. One of the most excellent merits of Sagnac typed sensors is their ultra-high sensitive ambient environment temperature changes because they have truly path-matched interference mode. Comparing to other optical fiber-based sensors that have to adopt some complex way to obtain high sensitivity, this quality helped Sagnac typed sensors gain a lot of attention. Apart from that, Sagnac loop sensors can be designed to get a higher sensitivity [58]. However, the widely adopted method of using broadband light with a Lyot-typed depolarizer [59] to control polarization fluctuations in Sagnac interferometric sensors might cause harm to the precision and SNR of the sensor. In addition, some of the Sagnac typed sensors could be quite complicated and difficult to fabricate [60]. So, researchers found that combining the FRL and Sagnac structure may solve these problems, and this method has been implemented recently.

In 2014, Wang et al. [61] found the high sensitivity and high-frequency response to ultrasonic wave signals which Sagnac interferometric sensors have are quite suitable for partial discharge (PD) acoustic sensing. However, they also noticed the depolarized light used in conventional Sagnac interferometers could bring in some disadvantages in sensitivity and SNR. By investigating former studies, they found the possibility of using a FRL based on an EDF amplifier (EDFA) working in chaotic mode to provide a degree of polarization (DOP) tunable light beam which can suppress the polarization fluctuation may solve the problem. So, they inserted a balanced Sagnac interferometer in to a FRL based on an EDFA to detect the acoustic properties in PD in power transformers. The balanced Sagnac interferometer played an important role, in their experiment, for command intensity noised eliminating and SNR enhancing, which ensured the experimental results finally reached a situation that the DOP of the laser beam can be tuned from 0.2% to 100% with nearly zero power fluctuation while the responding frequency of the sensor achieved 300 KHz. The structure of this sensor is well-designed and performed well in their experiment, however, the limitation of the experiment condition in their lab made it difficult for the research group to implement some test to explore the ability of this sensor, and some of the properties only can be theoretically deduced. Like the article said, the sensor needs more experiments in a more actual industrial environment.

**Figure 11:**
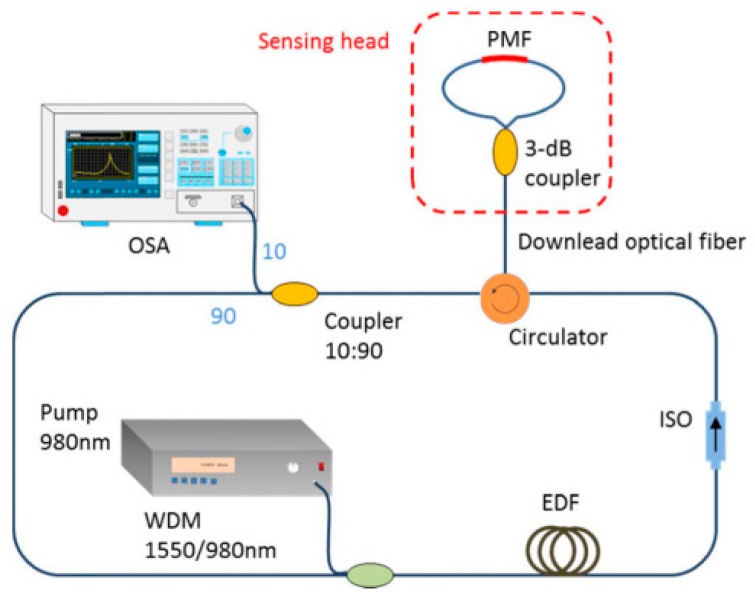
Schematic configuration of the multi-wavelength erbium-doped ring fiber laser based on an all fiber intrinsic Fabry–Perot interferometer.

Two year later, in 2016, Shi et al. [62] aimed into the temperature measuring domain, and spotted the trend that the designing of Saganc loop sensor tends to be more complex to achieve a higher sensitivity, so they tried to combine FRL with the Sagnac loop to simplify the structure of this kind of sensors, meanwhile, maintain or enhance the sensitivity of the sensor, and bring them a higher SNR and narrower 3-dB bandwidth. So, they produced a temperature sensor using FRL with a reflective Sagnac loop. The sensing head was fabricated by an insertion of the reflective Sagnac loop, which is constructed of a piece of polarization maintaining fiber (PMF), into the FRL through a downlead optical fiber and a 3-dB coupler. A circulator is also adopted into this system for coupling the propagating light signals. The structure of the sensing head is shown in Figure 11. The input light would get coupled in the circulator, propagate through the downlead optical fiber, and finally split into two light beams in the 3-dB coupler. These two beams would experience a phase dissimilation due to the birefringence of PMF, and start to interference with each other when passing through the 3-dB coupler again. To build the whole experimental system, the group set it up with a piece of EDF, a wavelength division multiplexing(WDM) coupler, an isolator(ISO), a reflective Sagnac loop, a coupler and an optical spectrum analyzer. The experimental setup of the FRL is also shown in Figure 11.

This sensor showed a high sensitivity of 1.739 nm/°C with a narrow bandwidth and a high signal-to-noise ratio when applied to detect the temperature of the environment. Comparing to other temperature based on FRL, this sensor did gain a higher sensitivity and maintain a simple structure. The special downlead optical fiber structure made enhanced the remote sensing ability of the sensor.

Another successful effort of mixing FRL and Sagnac interferometer together was made in 2015 by Sun et al. [63]. They demonstrated an in-line quasi-Saganc interferometer comb filter for tunable multi-wavelength fiber laser in 2015. The author adopted a polarizer, two fiber-coupled polarization controllers (PCs), a high-birefringence (Hi-Bi) fiber and a fiber mirror to build up their quasi-Sagnac interferometer. They finally tested and got the result that 14 lasing lines can be generated by the quasi-Sagnac interferometer with the SNR averaging from 0 dB to 40 dB in the wavelength range of 1500–1600 nm, and confirmed that this interferometer showed excellent qualities of multi-wavelength lasing lines operation, tunable ability, high SNR, and switchable ability when used as a comb filter.

An overview of these various types of sensors based on Sagnac is presented in Table 3.

### 3.4. FBG Typed Sensors

FBG is a type of distributed Bragg reflector constructed in a short segment of optical fiber that reflects particular wavelengths of light and transmits all others. This is achieved by creating a periodic variation in the RI of the fiber core, which generates a wavelength-specific dielectric mirror. Normally, a fiber grating with a period less than 1 µm can be considered as a FBG. FBG is a widely used structure in many domains of measurement. Its Bragg wavelength *λ*_B_ is given by:

(8) $$\lambda_{B}=2n_{\mathrm{eff}}\Lambda$$

where $n_{\mathrm{eff}}$ is the effective RI of the core mode while Λ is the grating pitch. Due to the effective RI is related to the RI of the ambient environment, the external RI changes will cause a shift of the Bragg wavelength. Besides, the surrounding temperature and strain will all change the $n_{\mathrm{eff}}$ and Λ. By detecting this shift, the external parameters can be measured.

For FBG typed sensors, they are durable, small, light-weighted, and they can also be easily embedded into other structures with nearly no impact on the properties of the structure, which makes them suitable for various applications [64,65]. In order to enhance the sensing accuracy, more and more researchers are interested in combining FBGs and FRLs. The FRL can bring high resolution for wavelength shift and high optical SNR against the noisy environments. Especially considering the fact that many of the FBGs using in some sensors are all experienced some chemical dealing, like etching, to make them more sensitive to the ambient environment but also harmful for the measurement resolution of these sensors, the insertion of FRL becomes a practical and cost-less way to solve this problem.

In the area of Acoustic Emission (AE) detection, FBG-based sensors are well-known for its small size, light weight, immune to electromagnetic interference. It is also normal to combine FBG structure and FRL structure in AE detection because it is easy to set the wavelength of lasers to the center of the linear range of the FBG reflection spectrum. As a research group working in this area, Han et al. [66] summarized the former works in this area and found it is hard to balance the contradiction between gaining a high sensitivity and remaining a low cost. In order to solve this problem, they focused on regular FBG due to its high sensitivity, simple structure, direct detection of the ultrasonic signal, and most important, low cost. Finally, they used a regular FBG and a tunable optical band-pass filter (TOBPF) to constitute a fiber-optic ultrasonic sensor in 2013. In their experimental system, the regular FBG, as the sensing element, was inserted into the laser cavity through a fiber-optic circulator. This simple structure and low cost of this sensor were its best merits, and it also showed potential when applied for structural health monitoring.

After a short while, Liu et al. [67] pursued this line of thought to improve the AE sensors. He also considered the influence that ambient temperature shifting bring to the FBGs, and produced a temperature-insensitive ultrasonic sensor system based on a FRL in 2014. They used a short and strong FBG as a sensing element, and a long and weak FBG as an adapting element while both of the FBGs were placed side-by-side in close proximity to make the sensor can adapt the ambient temperature drift. Both of the FBGs are incorporated into the FRL cavity. This sensor was confirmed to be able to measure ultrasonic steadily without affecting by the temperature changes.

In the same year of 2014, FBG-based sensors based on FRL did not just show its charm in the area of AE sensing. Shim et al. [68] also found its potential in strain and radiation dose sensing. Knowing that the EDF has high sensitivity to radiation, they did not just use the EDF-based fiber laser as a tool to enhance the SNR or narrow the 3-dB bandwidth of the sensor, but ingeniously using the EDF as a sensing probe for radiation dose detection. The real-time information of the irradiation dose can be recorded by analyzing the output power of the EDF ring laser. They also combined a FBG in this system as a strain sensing probe. So, the sensing scheme they proposed can be used for measurement of radiation dose and strain simultaneously. The sensitivities of the radiation and strain measurement reached 8.4 dB/km∙Gy and 0.81 pm/με, respectively. This research group contributed a lot to realize the multi-parameter measurement in combination of FBG and FRL though the strain sensitivity this sensor gained is not relatively high comparing to other strain sensors.

Another sensor based on similar physical concept, but used in RI sensing, was described by Shao et al. [69] in the year of 2016. They knew that the chemical etching process implemented on FBGs to make them sensitive for RI will do some harm to the resolution of these sensors, which limited the ability of accuracy sensing of this kind of sensors. One of the simplest ways to avoid reducing the measurement resolution of etched FBG typed sensors is to combine them with FRLs. Based on that thought, they presented a novel RI sensor with a dual-wavelength FRL. A FBG was etched for a cycle of 145 min in 30% HF solution and used as the RI sensing element. Another normal FBG which was used as temperature reference was incorporated with the cladding-etched FBG and inserted into the fiber ring cavity. This sensor has a narrow bandwidth comparing to other conventional FBG-based sensors, and finally reached a RI measurement resolution about 1.44 × 10^−5^ RIU within the range of 1.3330~1.4419. The sensor also gained a narrow 3-dB bandwidth (<0.015 nm) and a high SNR (~60 dB), which showed that the insertion of SNR did improve the sensing accuracy of the sensor. However, there still exist some room for the performance improvement of this sensor, and the temperature influence was not taken into consideration, which may somehow reduce the application of this sensor in some actual measurement condition.

**Figure 12:**
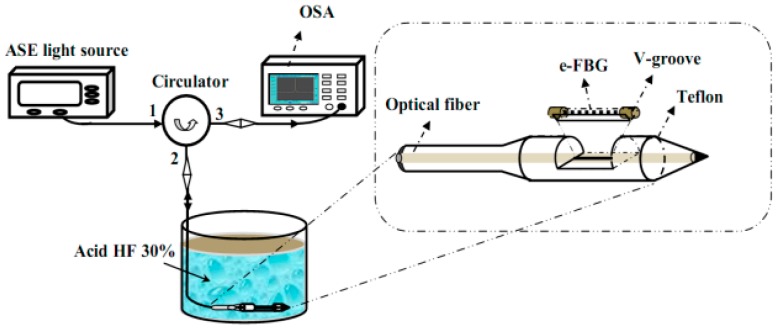
Setup of the fiber laser sensor system based on etched-Bragg grating sensing probe for determination of the low nitrate concentration in water.

In the next year, also the structure of combining etched FBGs and FRL structure but used to detect low nitrate concentration in a water environment, was proposed by Pham et al. [70]. They demonstrated characteristics of the fiber laser sensor system based on an etched-Bragg grating sensing probe. The sensing element is based on an etched fiber Bragg grating (e-FBG) which is formed by using the wet chemical etch-erosion and placed into a fiber cavity. A Teflon V-groove mount is chosen for its quality of non-reactant to HF and decreased mechanical vibration for e-FBG, and used as a protective mount for the fragile e-FBG. To detect the wavelength shift in the etching process, an experimental system is set up with an amplified spontaneous emission, an optical circulator and an optical spectrum analyzer. This setup and the designing of the fiber mount is shown in Figure 12.

In this system, the e-FBG is used as a reflector, and its reflection spectrum will be changed by the solution change of nitrate concentration. Due to this quality, the shift of Bragg wavelength can be monitored and analyzed in the OSA. This sensor showed good repeatability, rapid response and average sensitivity of 3.5 × 10^−3^ nm/ppm when detecting nitrate in water samples at a low concentration range with a SNR of 40 dB and 3-dB bandwidth of 0.02 nm. The results showed in their experiment indicated the ability of this sensor for determine the low nitrate concentration in outdoor environment.

**Figure 13:**
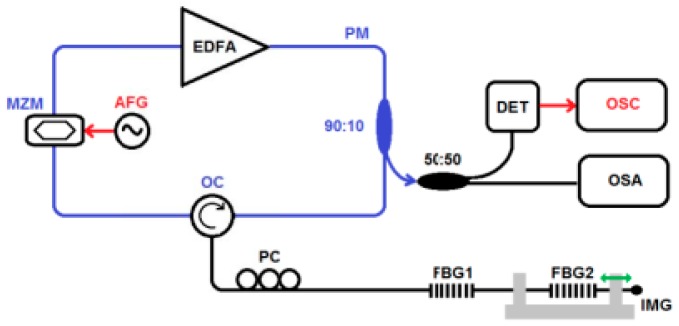
Actively mode-locked (AML) erbium-doped fiber amplifier (EDFA)-based fiber ring laser for fiber Bragg gratings (FBGs) interrogation.

Some other efforts were also made in researching combination of FBG and FRL. The cross sensitivity problem between temperature and strain is one of the major problems in the designing of the FBG-based sensors. The changes form the ambient temperature and strain will both cause the movement of the central wavelength of FBG. When FBG-based sensors are used for measurement, it is hard to tell whether the movement of the central wavelength is caused by strain change or temperature change. The insertion of FRL finds a new way to solve the problem. Osuch et al. [71] presented an actively mode-locked EDFA-based fiber ring laser interrogator, and it was tested that it can be used in simultaneous measurement of temperature and strain. Like what shows in Figure 13, they equipped a fiber loop of EDFA based FRL with an amplitude Mach–Zehnder modulator (MZM) which is driven by arbitrary function generator to construct an actively mode-locked (AML) configuration. In their reduction, the FBGs can be addressed respectively when the MZM is driven by a signal with a fundamental frequency of 4.38 MHz and 4.28 MHz, which means that the temperature and strain changes in the ambient environment can be detected by monitoring the changes in lasing wavelength. The AML technique they proposed also solved mode-competition problem in CW EDFA-based FRL.

An overview of these various types of sensors based on FBG is presented in Table 4.

### 3.5. Comparison and Analysis of Different Sensors

It is obvious that due to the excellent qualities of fiber laser ring in sensing domain, like the good performance of high optical SNR and narrow bandwidth which means that the fiber laser sensor would be applied to the sensing field with a long distance and high precision, it has been used widely in sensing different objects. To perfect its properties in certain different sensing domains, researchers did some work to make them more practical. Normally, most of the adjustment was made on the sensing head, which make the fiber laser ring sensors divide into four main types: MZI-typed, FPI typed, Sagnac-typed and FBG-typed. By comparing each type of sensors, we can find both of their merits and shortcomings. Qualities of different measurement parameters have been summarized in this article among different structure of sensing head. For example, the highest sensitivities of temperature sensing of MZI-based sensors, FPI-based sensors and Sagnac-based sensors are −1.747 nm/°C [28], 0.249 nm/°C [56], 1.739 nm/°C [62], respectively; the highest sensitivities of strain sensing among MZI-based sensors, and FBG-based sensors are 0.148 nm/με [38], 0.81 pm/με [68]. From the temperature sensitivity among different sensors, it can be seen that the MZI-based sensor and the Sagnac-based sensor can be designed to gain an extremely high sensitivity. However, the measurement range of these two sensors are limited in 25–31 °C and 30–40 °C. Both of them are quite narrow and remaining in a low degree. In contrast, the FPI-based sensor did not gain a relatively high sensitivity, but for trade-off, it can maintain a relatively wide measurement range of 30–−55 °C. However, the MZI-based sensors can also be designed to gain a wide measurement range like Liang et al. [26] did, though some necessary sacrifice are made on sensitivity. When it comes to the strain sensing, the MZI-typed sensor is also much more sensitive than the FBG-based sensor while the measurement range of the FBG-based sensor is much wider than the MZI-based sensor. Bur similar to Liang [26], the work of Liu [42] showed that MZI-based strain sensors could also be designed to gain a wider measurement range with sacrifice to the sensitivity.

In summary, among all the sensors based on FRL, MZI-typed sensors own a relatively bigger flexibility. Their merits of low cost, easy fabrication process and high stability make it possible for this kind of sensors to fit different situations. It can be designed to get a higher sensitivity with a lower measurement range, or it can also be designed to achieve an opposite goal. However, the inherently low sensitivity that MZI-based sensors have could also add the difficulty when researchers try to modify its structure to gain a higher sensitivity. The FPI-based sensors have higher sensitivity than normal-structured MZI-based sensors. Meanwhile, they can still maintain a simple structure, which means that researchers can do little modification on the traditional FPI structure to gain a considerable sensitivity, like what Zou [56] did. Moreover, the FPI-based sensors always have a miniature size, which makes it easier to combine it with a FRL. However, some of the Fabry–Perot typed sensors, like those used to detect chemical or biological objects, usually suffered a problem of its unsatisfied sensitivity. Sagnac-based sensors are sometimes designed 6with complex structure, however, they are also easily to gain a high sensitivity, like the work of Shi [62] showed. Sagnac-based sensors are also very robust and can be cascaded for several levels. But figuring a practical way to preserve polarization fluctuations in this kind of sensors is still a project that needs to be deeply studied. FBG-sensors are also widely used in many application fields due to that their measurement range can design to be large, which is also shown in the research of Shim et al. [68]. The small size of them makes them easily to be mixed with other structures, like FRL. Also, they have a good immunity to electromagnetic interference. However, some chemical processes conducted on FBG-based sensors may reduce the measurement resolution of the sensors, which made good room for FRL to fit in, and researchers already focus on this issue.

All in all, each type of the sensors has its own irreplaceable advantages and non-negligible shortages. When it comes to some certain areas of measuring, different types of sensors can be chosen to fit for the actual needs if we considered the distinguishing properties of every type of sensors carefully.

## 4. Existing Problems and Future Research Directions

Optical fiber sensors based on FRL has been widely used in different measuring areas. With the innovations implement on the sensing elements, this kind of sensors become more and more practical to the certain environment. However, there are still some problems need to be solve in the future research.

### 4.1. Parameter Optimization

In many papers, the authors paid a lot attention to renew the sensing head of the sensor. However, most of them ignored to optimize the parameter of the FRL structure. Some research about the properties of EDF points out that the output power of the EDF is related to length, doping concentration, coupling ratio and other parameters. Normally, the output power will experience a decrease followed by a rapid growth while the length or concentration or coupling ratio of EDF is increasing. So, it is obvious that introducing the discussing of the properties of EDF will help researchers to make their sensors more effective. In the future, a systematic method to determine the parameters of EDF in this kind of sensors should be explored and used to guide the designing process.

### 4.2. Multi-Parameter Measurement

Typical sensing structures are usually sensitive to several parameters. For example, the properties of MZIs can easily be changed by temperature [72], RI [73], humidity [74] and strain [75]. The FBG-based sensors also have the similar qualities. Though most of them have to experience some treatment on their basic structure like etching or tapering which may harm their strength, they still showed us the possibility of multi-parameter measurement. Considering the excellent qualities of FRL, the combination of the multi-parameter measurement methods with it is a prospective orientation. Actually, this idea has been implemented by some researchers like Liang [21], Kang [33], Cai [19] and some other researchers. However, we still have to admit that most of the researchers who studied the FRL for a long time neglected this area, which is such a pity. In the future, more effort should be made to figure out more multi-parameter measurement methods based on the FRL structure.

### 4.3. Remote-Distance Measurement

The combination of FRL and different kinds of sensing heads can definitely enhance the ability of the sensors in different ways. However, the dispose of the sensor in measuring process still needs more consideration. Most of the sensors based on FRLs tend to insert the sensing head into the FRL and place the whole device in the measuring environment. This arrangement may be convenient and low-cost, but the researchers ignored that most of the measuring environment is not friendly to the device. Excessive or prolonged exposure to these kinds of environment may cause some irreversible harm to the sensors. Therefore, in the future, researcher should pay more attention to this area, and figure out more methods to protect sensors away from device-unfriendly environment.

### 4.4. Mulitiplexing of the Sensors

Multiplexing capability is also a major quality to measure the performance of a sensor. Typically, the multiplexing ability of the sensors can be realized in two ways. The first one is to design a sensing system with several sensors using a same light source and checkout equipment. This plan can reduce the cost of the sensing system Another one is to design a sensing system which is compatible of the measurement of multipoint. This quality is important because some industrial devices in the real industrial environment are quite large, which means the monitoring of their whole inside condition should be multipoint. Considering the fact that the sensors with multiplexing ability can be deployed on the critical components of the large mechanical equipment to realize the functions, the necessity to endow FRL-based sensors the ability of multiplexing capacity is nonnegligible. To realize that goal, researchers can make some change on the sensing head. For example, the distributed FBG network shows good quality in multipoint condition monitoring [76], however, there is little work in trying to combine it with FRL. So, in the future, researcher can pay more attention on improving the multiplexing sensing ability of the FRL-based sensors, which will foreseeably enhance breadth of the application of this kind of sensors.

### 4.5. High Interrogation Speed

The different kinds of FRL-based sensors mentioned above are all using an OSA for optical readout. However, the interrogation technique based on OSA is rather slow and the resolution is also somehow limited. In the past few years, researchers have demonstrated that the optoelectronic oscillator (OEO) shows great quality in solving this problem. In 2013, Kong et al. [77] inserted a phase-shifted fiber Bragg grating (PS-FBG) into an OEO loop for transverse load sensing. In their experiment, the frequency interrogation allowed the sensor to gain an ultra-high speed. A year later, Kong et al. [78] combined the OEO loop and a FRL with a polarization-maintaining PS-FBG also for detecting transverse load. The combination provided the sensor a high accuracy measurement, and the microwave frequency interrogation also assured the sensor to gain an ultra-high speed. In 2017, the configuration of a dual-frequency OEO with two PS-FBGs were introduced for strain sensing. This sensor also showed good quality of getting a high speed and a high resolution [79]. In the future days, researchers focusing on FRL-based sensors could also try to abandon the OSA and insert the OEO, because the high interrogation speed and high resolution that this combination could bring may help this kind of sensors widen their application range.

## 5. Conclusions

In the last 10 years, a wide range of different sensing structures based on FRL has been proposed and carefully demonstrated. The excellent quality of its high optical SNR, high output power, low insertion loss and narrow bandwidth let it get more and more attention in the sensing domain, and be applied into various areas of sensing including temperature, strain, RI, liquid level, magnetic field, and so on. Different structures of sensing head and method were implemented on the basis of FRL to make it more targeting by researchers. This paper reviewed the structure, application, and properties of MZI typed sensors, FPI typed sensors, Sagnac typed sensors, FBG typed sensors and combined typed sensors. The MZI typed sensors based on FRL has been widely studied in recent years. This kind of sensors has already applied to measure many parameters, including temperature, RI, string, liquid level, magnetic field, curvature, gas concentration, displacement and bend. The MZI-based sensors are quite flexible, they can be used in the environment demanding high sensitivity or high measurement range. The small size that the FPI-based sensors got makes them easy to combine with FRL. They are used to detect humidity, Gas pressure and temperature. With simple modification on the typical FPI structure, the sensors can gain a considerable sensitivity and practical measurement range. Sagnac-based sensors combining with FRL are used to detect partial discharge and temperature. The FRL can be used as a low-cost light source in the Sagnac system to reduce polarization fluctuation. It is easy for Sagnac-based sensors to gain a high sensitivity, and the introducing of FRL can also reduce their structure complexity. Due to the ease of gaining a wide measurement range, FBG-based sensors are also used in many measurement application field, including acoustic emission, RI, radiation dose, strain and Nitrate concentration. The FRL shows good quality in enhancing the SNR that reduced by some chemical process in many of FBGs, which makes the combination of FRL and FBG quite valuable. The advantages and disadvantages of these kinds of sensing types are all summarized, and the existing problems and future research directions are also analyzed. High measurement resolution and increasing the ability on remote sensing to the sensors.

According to the review of the papers of the earlier researchers, it is obvious that sensors based on FRL can be very important in many domains of sensing. Combination of FRL and other sensing structures can bring high measurement resolution and increase on the ability of remote sensing to the sensors. With the development that the independent sensing head will experience in the future, it can be seen that more and more novel FRL-based sensing structures will be proposed with much wider application range.

## Figures and Tables

**Table 1:** Mach–Zehnder Interferometer typed sensors.

Ref.	Sensing Parameter	Structure of Sensing Head	Sensitivity	Measurement Range	3-dB Bandwidth	SNR	Author
[26]	Temperature	STCS fiber structure	10.8 pm/°C	8~80 °C	<0.12 nm	~40 dB	Liang
[27]		two-taper fiber	1089 pm/°C	55~70 °C	/	~50 dB	Martinez-Ríos
[19]		Offset Mach–Zehnder interferometer	0.049 nm/°C	22~50 °C	/	~60 dB	Cai
[28]		Liquid-filled PCF	−1.747 nm/°C	25~31 °C	<0.08 nm	∼55 dB	Yang
[29]	RI	SCS fiber structure	131.64 nm/RIU	1.333~1.3707	<0.03 nm	~50 dB	Liu
[30]		Bent fiber filter	124 nm/RIU	1.3259~1.3730	<0.3 nm	~52 dB	Zhang
[32]		Core-offset Mach–Zehnder interferometer	52.3 nm/RIU	1.334~1.370	<0.2 nm	~30 dB	Xing
[33]		FSF fiber structure	45.429 nm/RIU	1.333~1.363	<0.1 nm	~45 dB	Liu
[34]	String	PMS structure	2.1 pm/με	0–2100 με	<0.3 nm	~45 dB	Bai
[38]		All-fiber acoustooptic tunable filter	0.148 nm/με	0–160 με	<0.05 nm	~40 dB	Pei
[39]		Stretched abrupt tapered microfiber	4.443 nm/mε	0-7.0 mε	/	/	Chen
[40]		TCF	7.52 × 10^−4^ mW/µε	0–980 με	/	>40 dB	Kang
[41]		IMZI	103.5 pm/µm	20–80 μm	/	/	Jaddoa
[42]		FM-CRCF	0.81 pm/με	0–1467 με	<0.1 nm	~45 dB	Liu
[43]	Liquid level	Two taper structure	0.2294 nm/mm	0–20 mm	/	~50 dB	Zhang
[44]		SOCS	86.27 pm/nm	21–33 mm	<0.15 nm	~30 dB	Wang
[45]	Magnetic field	SNCS	12.05 pm/Oe	15.9–222.32 Oe	<0.19 nm	~40 dB	Bai
[49]		SNCS	52.1 pm/mT	0–120 Oe	<0.05 nm	~40 dB	Shi
[50]	Curvature	SNCS	−22.33 nm/m^−^^1^	0.2121–0.3463 m^−1^	<0.06 nm	>40 dB	Sun
[51]	Gas concentration	PCF	17.3 nW/ppm	0–400 ppm	/	/	Zheng
[52]	Displacement	SMF loop	227.5 pm/mm	0–30 mm	<0.7 nm	~52 dB	Liu
[53]	Bend	IMZI	~1.04 nm/m^−1^	0.8-2.0 m^−1^	<0.01 nm	~63 dB	Xiong

**Table 2:** Fabry–Perot Interferometer typed sensors.

Ref.	Sensing Parameter	Structure of Sensing Head	Sensitivity	Measurement Range	3-dB Bandwidth	SNR	Author
[54]	Gas pressure	FPI and Sagnac loop	−9.69nm/kPa	0.03–0.18 kPa	<0.02nm	~45 dB	Shi
[55]	Humidity	Humidity-sensitive FPI	0.202 dB/%RH	20% RH to 98% RH	<0.05nm	~45 dB	Shi
[56]	Temperature	FP air cavity between two SMFs	0.249 nm/°C	30~55 °C	<0.1514nm	~52 dB	Zou

**Table 3:** Sagnac Typed Sensors.

Ref	Sensing Object	Structure of Sensing Head	Sensitivity	Measurement Range	3-dB Bandwidth	SNR	Author
[61]	Partial Discharge	Balanced Sagnac interferometer	/	DOP = 0.2–95.8%	/	/	Wang
[62]	Temperature	Reflective Sagnac loop	1.739 nm/°C	30~40 °C	<0.05nm	~50 dB	Shi

**Table 4:** FBG typed sensors.

Ref.	Sensing Object	Structure of Sensing Head	Sensitivity	Measurement Range	3-dB Bandwidth	SNR	Author
[66]	Acoustic Emission	Regular FBG	/	/	/	/	Han
[67]	Acoustic Emission	Short and strong FBG	/	/	/	/	Liu
[68]	Radiation dose	EDF & FBG	8.4 dB/km∙G	0–300 Gy	/	/	Shim
	strain		0.81 pm/με	0–2000 με			
[69]	RI	Cladding-etched FBG	1.44 × 10^−5^ RIU	1.3330–1.4419 RIU	<0.015 nm	~60 dB	Shao
[70]	**Nitrate** concentration	Etched-Bragg grating	3.5 × 10^−3^ nm/ppm	0–80 ppm	<0.02 nm	~40 dB	Pham
